# Identification of a novel oncogenic mutation of *FGFR4* in gastric cancer

**DOI:** 10.1038/s41598-019-51217-6

**Published:** 2019-10-10

**Authors:** Takashi Futami, Tatsuya Kawase, Kenichi Mori, Makoto Asaumi, Rumi Kihara, Nobuaki Shindoh, Sadao Kuromitsu

**Affiliations:** Drug Discovery Research, Astellas Pharma Inc., Ibaraki, Japan

**Keywords:** Oncogenes, Gastric cancer

## Abstract

Gastric cancer remains one of the leading causes of cancer death worldwide. Despite intensive investigations of treatments over the past three decades, the poor prognosis of patients with unresectable advanced or recurrent gastric cancer has not significantly changed, and improved therapies are required. Here, we report the identification of an oncogenic mutation in *FGFR4* in a human gastric tumour that leads to constitutive activation of its product, FGFR4. The G636C-FGFR4 tyrosine kinase domain mutation was found in 1 of 83 primary human gastric tumours. The G636C mutation increased FGFR4 autophosphorylation, and activated FGFR4 downstream signalling molecules and enhanced anchorage-independent cell growth when expressed in NIH/3T3 cells. 3D-structural analysis and modelling of FGFR4 suggest that G636C destabilizes an auto-inhibitory conformation and stabilizes an active conformation, leading to increased kinase activation. Ba/F3 cell lines expressing the G636C-FGFR4 mutant were significantly more sensitive to ASP5878, a selective FGFR inhibitor, than the control. Oral administration of ASP5878 significantly inhibited the growth of tumours in mice engrafted with G636C-FGFR4/3T3 cells. Together, our results demonstrate that mutationally activated FGFR4 acts as an oncoprotein. These findings support the therapeutic targeting of FGFR4 in gastric cancer.

## Introduction

Gastric cancer is an aggressive cancer with poor prognosis and the fifth-most common cause of cancer-related death worldwide^1^. The number of survivors is low, because diagnosis is often determined during the late stages when symptoms first appear, which are accompanied by metastasis and chemoresistance^1^. Earlier treatment would be aided by detailed knowledge of the molecular characteristics of gastric cancer and the identification of new biomarkers. Most gastric cancers are adenocarcinomas, and arise and progress as a result of complex genetic and environmental interactions. The Cancer Genome Atlas (TCGA) identifies mutations that classify gastric cancer into the following subtypes: EBV-positive, microsatellite unstable/instability, genomically stable and chromosomal instability. The development of trastuzumab targeting ERBB2 is a successful example of translational genetic profiling and precision medicine applied to gastric cancer^2^. Such novel findings help uncover molecular mechanisms and identify effective therapeutic targets for gastric cancer.

The signalling pathways activated by fibroblast growth factor receptors (FGFRs) and their cognate ligands such as fibroblast growth factors (FGFs) play important roles in development, from early embryogenesis to the formation of organs^3,4^. FGFs and FGFRs are encoded by 18 and four genes, respectively, which are expressed by diverse cell types^3,4^. Upon ligand binding, FGFRs dimerize, autophosphorylate, and recruit adaptor proteins such as FGFR substrate 2^3,4^. These activate intracellular signalling pathways involved in cell growth, differentiation, and survival^3^. In gastric tissue, FGF10-triggered FGFR2 signalling controls stomach progenitor maintenance, morphogenesis and cellular differentiation during early epithelial growth before differentiation^5^. Approximately 1.2–9% of patients with gastric cancer harbour *FGFR2* amplifications associated with increased tumour cell proliferation^6^. Oncogenic gene alternations are present in all FGFR family members in human cancers. For example, *FGFR1* amplification occurs in breast, lung, gastric and bladder cancers^7–10^. *FGFR2* amplification, mutations and fusions occur in breast, liver, uterine, lung, gastric cancer^11–13^. FGFR3 mutations and fusions occur in bladder and lung cancers^12,14,15^. In contrast, *FGFR4* is infrequently mutated in cancers^16^. The oncogenic mutations of *FGFR4*, N535K/D and V550E/L, occur in rhabdomyosarcoma (RMS), although other oncogenic mutations are unknown^16^. The G388C mutation may be involved in tumour progression, although it is a polymorphism^17^.

Here, we describe the identification of a novel oncogenic mutation of FGFR4 (G636C) in gastric cancer. We show that G636C is a driver mutation that leads to enhanced sensitivity to a selective FGFR inhibitor. These results provide a rational basis for designing therapies that target FGFR4 in gastric cancer.

## Results

### Identification of FGFR4 TK-domain mutations in human gastric cancer

**Figure 1:**
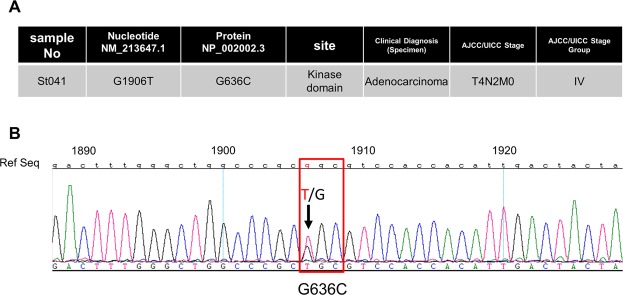
FGFR4 TK domain mutations in gastric cancer. (**A**) Site of mutation in the FGFR4 TK domain identified in a gastric cancer tissue specimen (n = 83). (**B**) Missense mutation in codon 636 (GGC –> TGC).

Oncogenic mutations of *FGFR4* occur in RMS, in which FGFR4 is highly expressed^16^. High FGFR4 expression correlates with tumour progression and survival in patients with gastric cancer^18^. We therefore hypothesized that FGFR4 plays an important role in gastric cancer and that mutations that activate the protein tyrosine kinase activity of FGFR4 promote an aggressive phenotype. Accordingly, we searched for activating *FGFR4* mutations of the tyrosine kinase domain in 83 gastric cancer tissue specimens. We identified a mutation in one sample (St041) at nucleotide position 1906 (NM_213647.1), which substitutes Gly with Cys at amino acid residue 636 (Fig. 1). Comparison with other clinical specimens revealed no clinical diagnostic features or stage that were unique to St041. This mutation is referenced in the Cancer Genome Atlas (TCGA: https://www.cancer.gov/about-nci/organization/ccg/research/structural-genomics/tcga), the Catalogue Of Somatic Mutations In Cancer (COSMIC; http://www.sanger.ac.uk/genetics/CGP/cosmic), dbSNP (http://www.ncbi.nlm.nih.gov/SNP) and the Human Gene Mutation Database (http://www.hgmd.cf.ac.uk/ac/index.php). G636C mutation was identified in oesophagogastric junctional adenocarcinoma and registered in the COSMIC database (COSMIC v89)^19^. This report did not include experimental analysis^19^. Further, human gastric cancer cell lines harbouring the G636C mutation are not listed in COSMIC (GRCh38 CELL_LINES v89).

### Structural analysis and modelling of FGFR4-G636C

**Figure 2:**
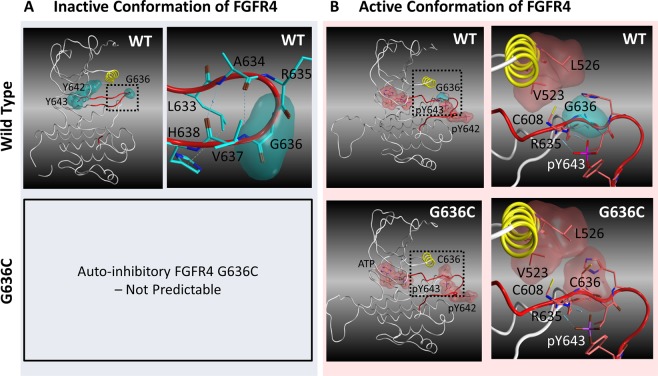
Three-dimensional structural models of catalytically inactive and active FGFR4. (**A**) X-ray structure of the kinase domain of FGFR4 in the auto-inhibitory conformation (PDB code: 4TYE^20^). The αC helix and A-loop are yellow and red, respectively. Blue and yellow dotted lines represent hydrogen bonds and the CH-π bond, respectively. The upper left panel shows an overview of the kinase domain, and the upper right panel shows the amino acid resides within 4.5 angstroms from G636. (**B**) Predicted 3D structure of the catalytically active conformation kinase domain. The left panels show overviews of the kinase domain, and the right panels show the amino acid resides within 4.5 angstroms from G636 or C636.

To investigate how G636C contributes to the activation of FGFR4, we systematically searched multiple x-ray crystallographic structures of FGFR4 deposited in the PDB. We found that G636 is located at a critical position that maintains the auto-inhibitory conformation (PDB code 4TYE)^20^. G636 resides in the activation loop of the FGFR4 TK domain (Fig. 2A). Specifically, the residues around G636 form a β-hairpin within an A-loop; moreover, only R635 and G636 reside on the tip of the turn between the two antiparallel β-strands (Fig. 2A). Through analysis of the dihedral angles of the backbone atoms around R635 and G636, we found that this structural motif is classified as a β-hairpin type I’ conformation, and Gly is the most frequent amino acid residue that forms this conformation^20–23^. In FGFR4, the backbone atoms at A634 and V637 form hydrogen bonds, and R635 and G636 make a sharp turn. The side-chains of L633 and H638 interact via a CH-π bond that may stabilize this conformation.

In contrast, we were unable to identify X-ray structures of phosphorylated catalytically active conformations of FGFR4. Homology modelling of phosphorylated G636C-FGFR4 in a catalytically active conformation indicated that C636 is located near the αC helix and engaged in hydrophobic interactions with V523 and L526 (Fig. 2B). Further, C608 is located within 4.5 angstroms from G636C. Because Cys is more hydrophobic than Gly, this result suggests that G636C strengthens this interaction in the active conformation.

### The G636C mutation promotes autophosphorylation of FGFR4 and phosphorylation of AKT and ERK

**Figure 3:**
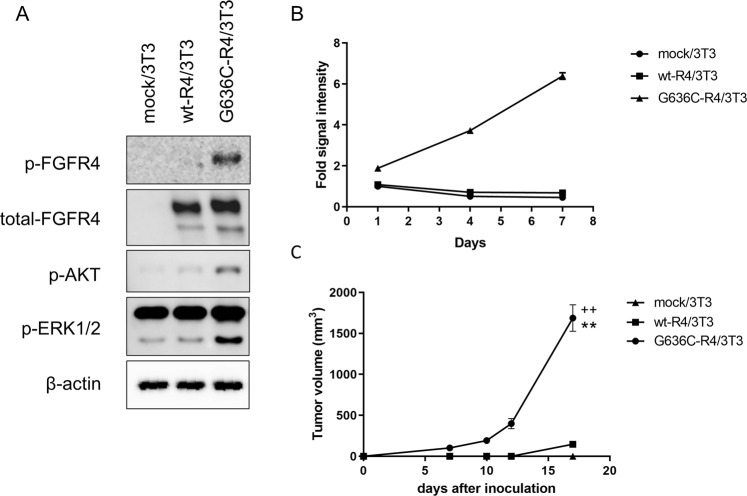
G636C-FGFR4 mutations transform 3T3 cells. (**A**) Expression and phosphorylation of G636C-FGFR4 and downstream signalling molecules. The blots cropped from different parts of gels are shown separately, indicated by the black frames. (**B**) Anchorage-independent growth of G636C-R4/3T3 cells. Experiment was performed in triplicate, and data are shown as mean ± SD. (**C**) Tumour growth after subcutaneous injection into mice of G636C-R4/3T3 cells. Data are shown as the mean ± SEM (n = 5). **P < 0.0001 compared with the mock/3T3 group (Student *t* test); ^++^P < 0.0001 compared with the wt-R4/3T3 group (Student *t* test).

To examine whether G636C mutation confers constitutive activation of FGFR4, we transduced NIH/3T3 cells with the wild-type human FGFR4 or G636C expression vector or empty vector (wt-R4/3T3, G636C-R4/3T3 and mock/3T3, respectively). Western blot analysis revealed that wt-R4/3T3 and G636C-R4/3T3 cells expressed comparable levels of FGFR4. Further, the levels of autophosphorylated FGFR4 and phosphorylated AKT and ERK in G636C-R4/3T3 cells were significantly higher compared with those of wt-R4/3T3 cells (Fig. 3A). These data indicate that G636C constitutively activates the TK activity of FGFR4.

### Cells expressing the FGFR4 mutant exhibit a malignant phenotype

We hypothesized that constitutive FGFR4 activation is associated with increased cell division. In support of this hypothesis, we found that in spheroid cell cultures, G636C-R4/3T3 underwent anchorage-independent proliferation at a significantly higher rate compared with that of wild-type FGFR4 (Fig. 3B). Consistent with these findings, subcutaneous injection of G636C-R4/3T3 cells into nude mice generated tumours with significantly larger volumes compared with those generated by wt-R4/3T3 cells (Fig. 3C).

We next transduced IL-3-dependent Ba/F3 cells^24^ with the FGFR4 expression vectors. Ectopic expression of G636C-FGFR4 conferred IL-3-independent survival and proliferation of Ba/F3 cells. Together, these data strongly support the conclusion that the G636C mutation converted FGFR4 into an oncoprotein.

### Antiproliferative effect of FGFR inhibitor on the cells expressing FGFR4-G636C

**Figure 4:**
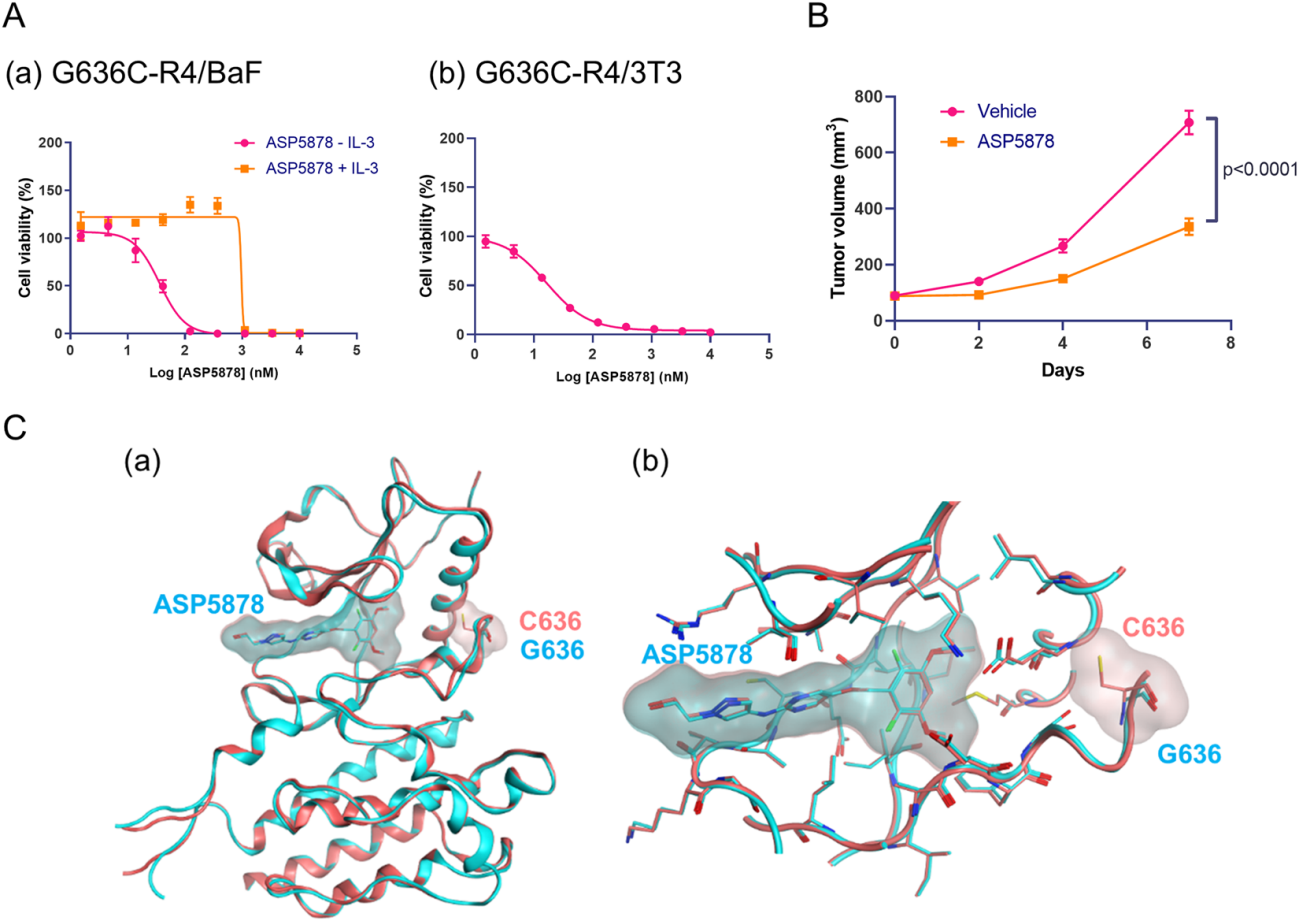
Antiproliferative effect of an FGFR inhibitor and 3D structures of FGFR4. (**A**) Antiproliferative effect of ASP5878 on (a) G636C-FGFR4/BaF3 and (b) G636C-FGFR4/3T3 cells. Three independent experiments were performed in triplicate, and representative data are shown as the mean ± SD. Geometric mean IC_50_ values and 95% confidence intervals (CIs) were determined from three independent experiments. (**B**) Mice engrafted with G636C-R4/3T3 cells were orally administered ASP5878 (3 mg/kg, once daily). Tumour volumes were measured, and data are shown as the mean ± SEM (n = 5). Tumour volumes on day 7 were compared between the ASP5878-treated and vehicle control groups (Student *t* test). (**C**) 3D models of FGFR4 wild-type and G636C with ASP5878. (a) Overview of 3D models of the kinase domain of FGFR4 (cyan, wild-type; pink, G636C). The heavy atoms of ASP5878, G636 or C636 are represented as sticks. (b) Close-up of the binding site of ASP5878.

Our results predict the activation of an oncogenic signaling pathway by *FGFR4-G636C*. To support this prediction, we treated G636C-R4/BaF3 and G636C-R4/3T3 cells with the selective FGFR inhibitor ASP5878^25,26^. G636C-R4/BaF3 cells were sensitive to ASP5878 (IC_50_, 34.4 nmol/L [95% CI: 24.6–48.2]) in the absence of IL-3 (Fig. 4A(a)). Specifically, in the presence of IL-3, the cells were approximately >10–fold less sensitive to ASP5878 (IC_50_ values > 370 nmol/L). In addition, G636C-R4/3T3 cells were sensitive to ASP5878 (IC_50_, 15.6 nmol/L [95% CI: 13.4–18.1]) (Fig. 4A(b)). This effect on growth was comparable to those of other ASP5878-sensitive cell lines, which are dependent on FGFR4 signalling^25,26^.

We next evaluated the anti-tumour activity of ASP5878 in a mouse subcutaneous xenograft model employing G636C-R4/3T3 cells. In this model, once-daily oral administration of ASP5878 (3 mg/kg) inhibited tumour growth by 60% (Fig. 4B) without body weight loss (data not shown). Three-dimensional structural modelling of wild-type and G636C-FGFR4 bound to ASP5878 indicated that the G636C mutation had no significant effect on binding ASP5878 compared with that of wild-type (Fig. 4C). These data indicate that G636C-FGFR4 may serve as a target for FGFR inhibitors now under evaluation in clinical trials^4,27,28^.

## Discussion

Recent success in molecularly-targeted therapies for cancer was made possible, in part, by identification of oncogenic gene alterations^2,29,30^. These discoveries led to the development of specific inhibitors having efficacious anti-tumour activities and reduced adverse effects compared to standard cytotoxic drugs. Examples include erlotinib and crizotinib, which are specific for mutant EGFRs and ALK/ROS-driven lung cancers, respectively^29,30^. Although the number of therapies targeted to driver oncogenes of gastric cancer is limited, a humanized monoclonal antibody against HER2 (trastuzumab) was approved by the United States Food and Drug Administration (USFDA) for the treatment of patients with metastatic gastric cancer that overexpress HER2^1,2^. Moreover, nine selective FGFR inhibitors are now in development for the treatment for gastric cancer with *FGFR2* amplification^6^.

Here, we identified a novel oncogenic mutation (G636C) of *FGFR4* in a primary gastric cancer. Computational analysis shows that G636C activated FGFR4 by disrupting the auto-inhibitory conformation and stabilizing the catalytically active conformation of its A-loop (Fig. 2). Functional analysis showed that NIH/3T3 fibroblasts ectopically expressing G636C-FGFR4 exhibited a malignant phenotype (Fig. 3) and that G636C acted as a driver mutation that led to enhanced sensitivity to the FGFR inhibitor ASP5878 (Fig. 4). The sensitivity of G636C-FGFR4-transformed Ba/F3 cells and NIH/3T3 cells to ASP5878 was comparable to that of other ASP5878-sensitive cell lines (Hep3B2.1-7, HuH-7, and JHH-7), which are dependent on signalling through the FGFR4^11,25,26^. Once-daily oral administration of ASP5878 significantly inhibited the growth of tumours in mice engrafted with G636C-FGFR4/3T3 cells (Fig. 4).

We identified G636C-FGFR4 in only one of 83 gastric tumour specimens. In the absence of studies of larger numbers of gastric tumours from diverse sources, this finding indicates that G636C-FGFR4 is a minor mutation in gastric cancer. G636C-FGFR4 occurs in oesophagogastric junctional adenocarcinoma^19^. Further, mutations corresponding to G636-FGFR4 include D647N-FGFR1 in lung cancer, D650H-FGFR2 in stomach cancer, D650Y-FGFR2 in endometrial cancer and D641N-FGFR3 in bladder cancer^31–34^. In particular, the tyrosine kinase activity of FGFR3-D641N is increased compared with that of the wild-type isoform^35^. Analysis by COSIC using Hidden Markov Models (FATHMM) software predicted that these mutations are pathogenic (FATHMM score >0.95). Although further investigation is required to confirm the oncogenicity of these mutations, these observations suggest that G636C-FGFR4 and its corresponding mutations in other members of the FGFR family may serve as common therapeutic targets across tumour type. For example, the USFDA approved a TRK inhibitor for adult and paediatric patients with cancers that harbour oncogenic *NTRK* fusions involving *NTRK1*, *NTRK2* or *NTRK3*, which are present at low frequencies (commonly <1%) in diverse tumour types^36^.

The FGFR-specific inhibitors NPV-BGJ398, AZD4547 and JNJ-42756493 are under development for treating lung and breast cancers with *FGFR1* amplification, gastric cancer with *FGFR2* amplification, cholangiocarcinomas with an *FGFR2* fusion, and urothelial cancers with *FGFR3* alterations^28^. The FGFR4-specific kinase inhibitors BLU9931 and FGF401 are now in clinical trials that include patients with HCC^4,27^. The development of these FGFR inhibitors may provide effective therapies for G636C-positive gastric cancer. For example, JNJ-42756493, a potent inhibitor of the tyrosine kinase activities of the four FGFR family members, may be beneficial for treating gastric cancer with *FGFR2* amplification and those that harbour the G636C-FGFR4 mutation^37^.

FGFRs exist in an equilibrium between active and inactive conformations, and gain-of-function mutations shift the equilibrium to the active conformation^38^. Therefore, we evaluated potential mechanisms that may explain how G636C increases the kinase activity of FGFR4 (Supplementary Fig. S2) as follows: (1) destabilization of the inactive conformation and (2) stabilization of the active conformation, which shift the equilibrium to the active conformation. We first focused on the destabilization of the inactive conformation, because we found that G636 forms a two-residue β-hairpin type-I’ conformation in the auto-inhibitory conformation (Fig. 2A). Moreover, Gly resides at this position in most type-I’ β-hairpin structures^20–23^, suggesting that FGFR4-G636C likely disrupts this β-hairpin structure. Such a conformational change may increase the dynamics of the activation loop and increase the probability of exposing Y642 and Y643 to solvent. In the auto-inhibitory conformation, Y642 and Y643 prevent the insertion of ATP into the binding pocket, leading to inhibition of kinase activity^20^. Moreover, the phosphorylation of the two tyrosine residues is required to activate the FGFR4 kinase^39^. Therefore, G636C may increase the exposure of the two Tyr residues to solvent, increasing the probability that ATP binds the pocket and the two tyrosine residues are phosphorylated.

We further considered the possibility that G636C increases the population of active conformers of FGFR4 via stabilization of the active conformation. Unfortunately, we were unable to identify the active conformation of FGFR4 in the PDB. Therefore, we predicted the 3D structure of phosphorylated G636C-FGFR4 and investigated the interaction network surrounding the mutated residue (Fig. 2B). The results reveal that C636 is most likely positioned near V523, L526 and C608. V523 and L526 reside in the αC helix, and C608 in the β-strand contributes to the formation of the β-sheet within a region of the A-loop. Analyses of X-ray structures and NMR data of mutated FGFRs indicate that critical common mechanisms for the activation of FGFR kinase activity are as follows: “molecular brake”, “DFG-latch”, “A-loop plug” and “αC tether”^38^.

The mechanism of the “αC tether” involves a stabilization of the active conformation through interaction between the activation loop and a catalytically critical αC helix. This mechanism was demonstrated using variants of FGFR2-D650, which interact with M537 and M540 in the αC helix in the active conformation^42^. The study found that the more hydrophobic residues D650A, D650L, D650V and D650I increased FGFR2 kinase activity by 3-fold to 19-fold, whereas D650G, which lacks a side-chain that interacts with M537 and M540, decreases kinase activity^38^. Further, analysis of the variants M537 and M540 revealed a correlation between hydrophobicity at these positions and kinase activity^38^. Accordingly, D650 contributes to kinase activation by stabilizing the active conformation through formation of a hydrophobic interaction with the catalytically important αC helix^38^. Moreover, D650, M537 and M540 of FGFR2 correspond to G636, V523 and L526 of FGFR4, respectively.

Therefore, our present modelling of the active form of FGFR4 is consistent with these observations, suggesting that the effect of the G636C mutation on kinase activity is explained by the “αC tether” mechanism. Our model predicts that G636C similarly interacts with V523 and L526 in the αC helix, possibly strengthening the hydrophobic interaction compared with that of wild-type G636. However, in the FGFR3-D641 variants, equivalent to D650 in FGFR2, D641N and D641G increase kinase activity^35^. This observation suggests that another mechanism activates the kinase. Further, our results indicate that FGFR4-C608 resides close to G636C, suggesting that G636C forms a disulfide bond with C608 to stabilize the active conformation. Further studies are required to reveal the mechanism of the effect of this mutation on the kinase activities of FGFRs. Moreover, our findings indicate that FGFR kinase inhibitors such as ASP5878 may serve as therapeutic agents for gastric cancer.

## Methods

### Clinical specimens

RNA specimens (n = 83) purified from primary gastric tumours were obtained from Asterand Bioscience. Written informed consent was obtained from all patients. Patient anonymity was ensured, and the study was approved by the Institutional Review Committees at Astellas Pharma, Inc. (Astellas). Experiments were performed in accordance with Astellas’ guidelines.

### Nucleotide sequencing

Total RNAs were reverse-transcribed using SuperScript III reverse transcriptase (Thermo Fisher Scientific). The tyrosine kinase (TK) domain of *FGFR4* was amplified from cDNA templates using PCR with the forward primer (5′-CACTGTGGCCGTCAAGATGC-3′) and reverse primer (5′-TGCTGGTTTTCTTATAGTAGTCAA-3′). Nucleotide sequencing of PCR products was performed using Big Dye Terminator chemistry (Applied Biosystems) and the forward primer (5′-ATGCTCAAAGACAACGCCTCTGAC-3′). Sequences were analysed using SeqMan Pro software (DNASTAR, Lasergene). Experiments were carried out in accordance with Astellas’ guidelines.

### Plasmids and cell lines

Human wild-type *FGFR4* (wt-R4, NM_213647.1) and G636C-*FGFR4* (G636C-R4) were cloned into the pMXs-Puro retroviral vector (Cell Biolabs). NIH/3T3 cells and Ba/F3 cells were obtained from the ATCC and RIKEN Bioresource Center, respectively. Although the cell lines used in this study were not authenticated in our laboratory, they were purchased from providers of authenticated cell lines and stored at early passages in a central cell bank at Astellas. Mycoplasma testing was performed using PCR. The experiments were conducted using low-passage cultures of these stocks. NIH/3T3 cells were cultured in DMEM medium supplemented with 10% heat-inactivated FBS. Ba/F3 cells were maintained in RPMI-1640 medium supplemented with 10% heat-inactivated FBS and mouse IL-3 (10 ng/mL). Wt-R4- or G636C-R4- or mock-infected NIH/3T3 cells and G636C-R4-Ba/F3 cells were generated by Astellas. The pMXs-Puro retroviral vector containing the wt-R4 or G636C-R4 gene or empty vector was transfected into Platinum-E cells using FuGENE HD transfection reagent (Roche Diagnostics) to produce virus stocks. NIH/3T3 and Ba/F3 cells were then infected with viruses with genomes harbouring each FGFR4 construct or control virus. Stable transfectants were obtained and maintained under selection pressure using puromycin at 1.5 µg/mL. G636C-R4-Ba/F3 cells were cultured in RPMI-1640 as described above but without IL-3. Experiments were carried out in accordance with institutional guidelines approved by Astellas.

### *In vitro* anchorage-independent growth assay

Mock/3T3, wt-R4/3T3 and G636C-R4/3T3 cells (1000 cells per well) were plated in DMEM supplemented with 10% heat-inactivated FBS in 96-well Sumilon Celltight spheroid plates (Sumitomo Bakelite) and then incubated at 37 °C in an atmosphere containing 5% CO_2_. The number of viable cells was determined using the CellTiter-Glo Luminescent Cell Viability Assay (Promega) on days 1, 4 and 7. Experiments were carried out in accordance with Astellas’ guidelines.

### *In vitro* growth assay

G636C-R4/BaF cells (1000 cells per well) were plated in RPMI-1640 supplemented with 10% heat-inactivated FBS with or without IL-3 (10 ng/mL) in 96-well plates. G636C-R4/3T3 cells (2000 cells per well) were plated in DMEM supplemented with 10% heat-inactivated FBS in PrimeSurface96U plates (Sumitomo Bakelite). The cells were treated with ASP5878 at 0–10,000 nM (3-fold serial dilutions, 10 concentration points) for 4 or 5 days, and the number of viable cells was determined using the CellTiter-Glo Luminescent Cell Viability Assay (Promega). The IC_50_ value of ASP5878 was calculated using nonlinear regression analysis with the Sigmoid–Emax model, and geometric mean IC_50_ values and 95% confidence intervals (CIs) were calculated from three individual experiments. Microsoft Excel (Microsoft) and GraphPad Prism (GraphPad Software) were used for data analysis. Experiments were performed in accordance with Astellas’ guidelines.

### Western blotting

NIH/3T3 cells were lysed with cell lysis buffer containing phosphatase and protease inhibitors, and the levels of FGFR4, phospho-FGFR4, phospho-ERK1/2, phospho-AKT and β-actin were determined using immunoblotting. The antibodies were as follows: anti-FGFR4 from Santa Cruz Biotechnology, Inc. (Dallas, TX); and anti-phospho-FGFR (Tyr653/654), anti-phospho-ERK (Thr202/Tyr204), anti-phospho-AKT, anti-β-actin and HRP-conjugated anti-rabbit IgG from Cell Signaling Technology (Danvers, MA). All antibodies were diluted 1/2000 for use. Proteins of interest were visualized by enhanced chemiluminescence using the Western Lightning Plus-ECL (PerkinElmer) and detected with LAS-4000 (GE Healthcare). Cell lysis buffer was purchased from Cell Signaling Technology, phosphatase inhibitor cocktail from Thermo Scientific (Rockford, lL) and protease inhibitor cocktail from Roche Diagnostics (Mannheim, Germany). Experiments were performed in accordance with Astellas’ guidelines.

### Structural analysis and modelling

The amino acid sequence of the kinase domain of wild-type FGFR4 was obtained from the UniProt database (UniProt ID: P22455, isoform 1). Substituting G636 with Cys generated the mutant G636C-FGFR4. The coordinates of the auto-inhibitory conformation of FGFR4 are registered in the protein database (PDB) (PDB ID: 4TYE)^20^. An X-ray structure of the phosphorylated catalytically active form of FGFR4 with ATP or ATP analogues is not available, to our knowledge. Therefore, the coordinates of the active conformation of G636C-FGFR4 were modelled according to the phosphorylated- and ATP analogue-bound active form of FGFR1 (PDB ID: 3GQI)^40^. The coordinates of 3GQI were used as a template for homology modelling of the active form of FGFR4 using the modelling software MOE (Chemical Computing Group). Phosphate moieties of Y642 and Y643 were added after homology modelling, and energy minimization of the coordinates of these residues was performed to relax the coordinates. Further energy minimization including R635 and G636C was performed, because an Arg residue corresponding to R635 forms hydrogen bonds with a phosphorylated Tyr residue corresponding to Y643 of other FGFR isoforms^6,41,42^.

The coordinates of ASP5878-bound wild-type FGFR4 were modelled according to the coordinates of wild-type FGFR4 bound to the FGFR kinase inhibitor BLU9931 (PDB ID: 4XCU)^22^, whose chemical structure is similar to that of ASP5878. Sequentially substituting or removing atoms and bonds of BLU9931 in 4XCU from the atoms binding to the back of the binding pocket generated the initial coordinates of FGFR4 bound to ASP5878. Repetitive optimization calculations of the coordinates were performed using energy minimization, and the atoms of the proteins were fixed using MOE. Finally, the coordinates of the residues within 4.5 angstroms from ASP5878 were repeatedly optimized until the energies converged. The coordinates of ASP5878-G636C-FGFR4 were introduced using homology modelling by MOE that were based on the coordinates of wild-type ASP5878-FGFR4. During the homology modelling, the coordinates of the atoms were initially assigned according to the template structure and finally optimized to minimize the energy level, which reflected the effect of the mutated residue on the coordinates near the residue as well as those of the binding pocket. To visualise the molecules, hydrogen atoms were added using Protonate3D included in MOE.

### ASP5878

ASP5878 (Astellas Pharma Inc^43^) was synthesized in our laboratory. Experiments were performed in accordance with Astellas’ guidelines. ASP5878 was dissolved in dimethyl sulfoxide or 0.5% methylcellulose (MC) for *in vitro* or *in vivo* experiments, respectively.

### Xenograft model

The Institutional Animal Care and Use Committee of Astellas approved the experimental protocols for using animals. Astellas Pharma Inc., Tsukuba Research Center is accredited by the Association for Assessment and Accreditation of Laboratory Animal Care International. Experiments were carried out in accordance with Astellas’ guidelines. Four-week-old male nude mice (CAnN.Cg-Foxn1nu/CrlCrlj [nu/nu]) were obtained from Charles River Japan.

### *In vivo* xenograft study of the tumourigenicity of cells expressing G636C-FGFR4

Mock/3T3, wt-R4/3T3 and G636C-R4/3T3 cells were cultured in DMEM supplemented with 10% heat-inactivated FBS, and 0.1 ml of 3 × 10^6^ cells mixed with Matrigel/PBS ([Matrigel:PBS = 1:1]) was subcutaneously inoculated into the flanks of mice. Tumour volume was calculated as follows: V = length × width^2^ × 0.5. Matrigel was purchased from Corning Incorporated (Life Sciences).

### *In vivo* xenograft study on the antitumour activity of ASP5878

G636C-R4/3T3 cells were subcutaneously inoculated into the flanks of mice (5 × 10^6^ cells/0.1 mL [matrigel:PBS = 1:1]/mouse). Seven days after inoculation, mice with tumours were divided such that the mean tumour volume was similar among the groups on the first day (day 0) of treatment. ASP5878 (3 mg/kg) was orally administered once daily for 8 days. Tumour diameters were measured using a caliper on days 0, 2, 4, and 7, and tumour volume was determined by calculating the volume of an ellipsoid as follows: length × width^2^ × 0.5. Body weight was measured using a standard balance.

### Statistical analysis

The values for the mouse xenograft model are expressed as the mean ± SEM. Differences between groups were analysed using the Student *t* test. P < 0.05 was considered statistically significant. Microsoft Excel (Microsoft) and GraphPad Prism (GraphPad Software) were used for data analysis.

## Supplementary information

Supplementary information

## Data Availability

All data generated or analysed during this study are included in this published article (and its Supplementary Information Files).

## References

[1] Liu X, Meltzer SJ (2017). Gastric Cancer in the Era of Precision Medicine. Cell Mol Gastroenterol Hepatol.

[2] Bang YJ (2010). Trastuzumab in combination with chemotherapy versus chemotherapy alone for treatment of HER2-positive advanced gastric or gastro-oesophageal junction cancer (ToGA): a phase 3, open-label, randomised controlled trial. Lancet.

[3] Beenken A, Mohammadi M (2009). The FGF family: biology, pathophysiology and therapy. Nat Rev Drug Discov.

[4] Hierro C, Rodon J, Tabernero J (2015). Fibroblast Growth Factor (FGF) Receptor/FGF Inhibitors: Novel Targets and Strategies for Optimization of Response of Solid Tumors. Semin Oncol.

[5] Nyeng P, Norgaard GA, Kobberup S, Jensen J (2007). FGF10 signaling controls stomach morphogenesis. Dev Biol.

[6] Hierro C (2017). Targeting the fibroblast growth factor receptor 2 in gastric cancer: promise or pitfall?. Ann Oncol.

[7] Luo J (2017). An mRNA Gene Expression-Based Signature to Identify FGFR1-Amplified Estrogen Receptor-Positive Breast Tumors. J Mol Diagn.

[8] Weiss J (2010). Frequent and focal FGFR1 amplification associates with therapeutically tractable FGFR1 dependency in squamous cell lung cancer. Sci Transl Med.

[9] Peifer M (2012). Integrative genome analyses identify key somatic driver mutations of small-cell lung cancer. Nat Genet.

[10] Campbell JD (2016). Distinct patterns of somatic genome alterations in lung adenocarcinomas and squamous cell carcinomas. Nat Genet.

[11] Turner N (2010). Integrative molecular profiling of triple negative breast cancers identifies amplicon drivers and potential therapeutic targets. Oncogene.

[12] Wu YM (2013). Identification of targetable FGFR gene fusions in diverse cancers. Cancer Discov.

[13] Tchaicha JH (2014). Kinase domain activation of FGFR2 yields high-grade lung adenocarcinoma sensitive to a Pan-FGFR inhibitor in a mouse model of NSCLC. Cancer Res.

[14] Comprehensive molecular characterization of urothelial bladder carcinoma (2014). Nature.

[15] Gao Q (2018). Driver Fusions and Their Implications in the Development and Treatment of Human Cancers. Cell Rep.

[16] Taylor JGT (2009). Identification of FGFR4-activating mutations in human rhabdomyosarcomas that promote metastasis in xenotransplanted models. J Clin Invest.

[17] Morimoto Y (2003). Single nucleotide polymorphism in fibroblast growth factor receptor 4 at codon 388 is associated with prognosis in high-grade soft tissue sarcoma. Cancer.

[18] Ye Y (2010). The fibroblast growth factor receptor-4 Arg388 allele is associated with gastric cancer progression. Ann Surg Oncol.

[19] Chong IY (2013). The genomic landscape of oesophagogastric junctional adenocarcinoma. J Pathol.

[20] Lesca E, Lammens A, Huber R, Augustin M (2014). Structural analysis of the human fibroblast growth factor receptor 4 kinase. J Mol Biol.

[21] Milner-White EJ, Poet R (1986). Four classes of beta-hairpins in proteins. Biochem J.

[22] Blundell TL, Sibanda BL, Sternberg MJ, Thornton JM (1987). Knowledge-based prediction of protein structures and the design of novel molecules. Nature.

[23] Gunasekaran K, Ramakrishnan C, Balaram P (1997). Beta-hairpins in proteins revisited: lessons for de novo design. Protein Eng.

[24] Kong, K., Ng, P. K. & Scott, K. L. In *Oncotarget* Vol. 8 35488-35489 (2017). 10.18632/oncotarget.17828 PMC5482591 28500277

[25] Futami T (2017). ASP5878, a Novel Inhibitor of FGFR1, 2, 3, and 4, Inhibits the Growth of FGF19-Expressing Hepatocellular Carcinoma. Mol Cancer Ther.

[26] Kikuchi A (2017). ASP5878, a selective FGFR inhibitor, to treat FGFR3-dependent urothelial cancer with or without chemoresistance. Cancer Sci.

[27] Hagel M (2015). First Selective Small Molecule Inhibitor of FGFR4 for the Treatment of Hepatocellular Carcinomas with an Activated FGFR4 Signaling Pathway. Cancer Discov.

[28] Sandhu DS, Baichoo E, Roberts LR (2014). Fibroblast growth factor signaling in liver carcinogenesis. Hepatology.

[29] Pao W (2004). EGF receptor gene mutations are common in lung cancers from “never smokers” and are associated with sensitivity of tumors to gefitinib and erlotinib. Proc Natl Acad Sci USA.

[30] Malik SM (2014). U.S. Food and Drug Administration approval: crizotinib for treatment of advanced or metastatic non-small cell lung cancer that is anaplastic lymphoma kinase positive. Clin Cancer Res.

[31] Kakiuchi M (2014). Recurrent gain-of-function mutations of RHOA in diffuse-type gastric carcinoma. Nat Genet.

[32] Jeske YW (2017). FGFR2 mutations are associated with poor outcomes in endometrioid endometrial cancer: An NRG Oncology/Gynecologic Oncology Group study. Gynecol Oncol.

[33] Lott S (2009). FGFR3 and TP53 mutation analysis in inverted urothelial papilloma: incidence and etiological considerations. Mod Pathol.

[34] Katoh M (2019). Fibroblast growth factor receptors as treatment targets in clinical oncology. Nat Rev Clin Oncol.

[35] Patani H (2016). Landscape of activating cancer mutations in FGFR kinases and their differential responses to inhibitors in clinical use. Oncotarget.

[36] Cocco E, Scaltriti M, Drilon A (2018). NTRK fusion-positive cancers and TRK inhibitor therapy. Nat Rev Clin Oncol.

[37] Perera TPS (2017). Discovery and Pharmacological Characterization of JNJ-42756493 (Erdafitinib), a Functionally Selective Small-Molecule FGFR Family Inhibitor. Mol Cancer Ther.

[38] Chen, H. *et al*. Elucidation of a four-site allosteric network in fibroblast growth factor receptor tyrosine kinases. *Elife* **6**, 10.7554/eLife.21137 (2017). 10.7554/eLife.21137 PMC5293489 28166054

[39] Wang J, Yu W, Cai Y, Ren C, Ittmann MM (2008). Altered fibroblast growth factor receptor 4 stability promotes prostate cancer progression. Neoplasia.

[40] Bae JH (2009). The selectivity of receptor tyrosine kinase signaling is controlled by a secondary SH2 domain binding site. Cell.

[41] Chen H (2007). A molecular brake in the kinase hinge region regulates the activity of receptor tyrosine kinases. Mol Cell.

[42] Chen H (2008). A crystallographic snapshot of tyrosine trans-phosphorylation in action. Proc Natl Acad Sci USA.

[43] Kameda, M. *et al.* Astellas Pharma Inc., Kotobuki Pharm Co., Ltd, assignee. Nitrogen-containing aromatic heterocyclic compound. WO 2013/129369 A1. 2013 Sep 6.

