# Supplementary material for: Identification of a novel oncogenic mutation of FGFR4 in gastric cancer
Source: Sci Rep. 2019 Oct 10;9:14627. doi: 10.1038/s41598-019-51217-6 (PMC6787178; doi:10.1038/s41598-019-51217-6)
Supplement: Supplementary file 1 — Supplementary information [file 41598_2019_51217_MOESM1_ESM.pdf]

# Identification of a novel oncogenic mutation of *FGFR4* in gastric cancer

Takashi Futami*, Tatsuya Kawase*, Kenichi Mori, Makoto Asaumi, Rumi Kihara, Nobuaki Shindoh, and Sadao Kuromitsu

Drug Discovery Research, Astellas Pharma Inc., Ibaraki, Japan

**Corresponding Author:** Takashi Futami

Drug Discovery Research, Astellas Pharma Inc., 21 Miyukigaoka Tsukuba-shi, Ibaraki 305-8585, Japan

Tel: +81-3-3244-3086; Fax: +81-3-3244-0448

E-mail: takashi.futami@astellas.com

**Supplementary Information**

**Fig. S1 Full gel images of Fig. 3A**

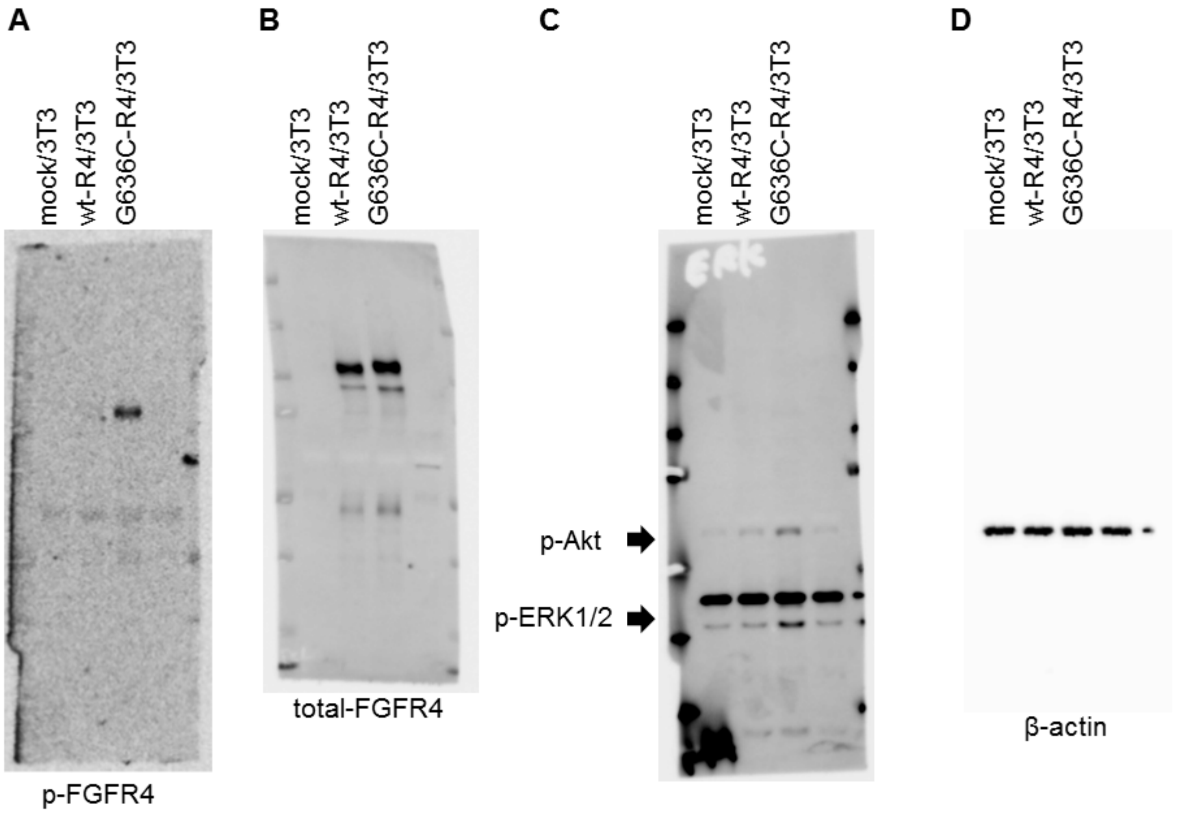


Expression and phosphorylation of FGFR4 and downstream signalling molecules A) phospho-FGFR4, B) FGFR4, C) phospho-ERK1/2, phospho-AKT and D) β-actin

**Fig. S2. Effects of the FGFR4 G636C mutation on the equilibrium between active and inactive conformations.**

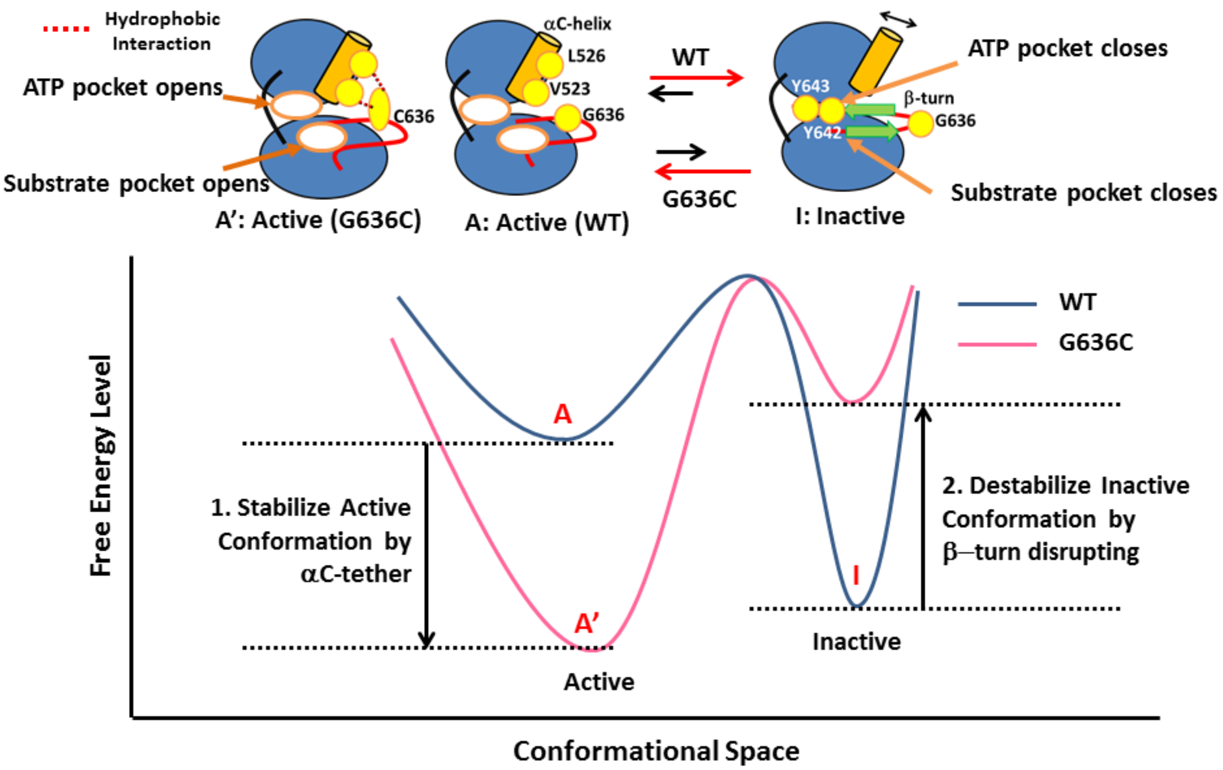


The kinase domain of FGFR4 is in equilibrium between active and inactive conformations. The probability of the formation of these conformations depends on the free energy level. The lower the free energy of a conformation, the higher its probability. The G636C mutation shifts the equilibrium to the active conformation through the following effects: (1) stabilization and lowering the free energy level of the active conformation through hydrophobic interactions among C636, V523, and L526 (αC-tether), (2) destabilization and increasing the free energy of the inactive conformation by distrupting the β-turn.
